# The Mystical Experience Questionnaire 4-Item and Challenging Experience Questionnaire 7-Item

**DOI:** 10.1089/psymed.2023.0046

**Published:** 2024-03-12

**Authors:** Justin C. Strickland, Albert Garcia-Romeu, Matthew W. Johnson

**Affiliations:** Department of Psychiatry and Behavioral Sciences, Johns Hopkins University School of Medicine, Baltimore, Maryland, USA.

**Keywords:** challenging experience, LSD, mystical experience, psilocybin, psychedelics

## Abstract

**Background:**

The Mystical Experience Questionnaire (MEQ-30) and Challenging Effects Questionnaire (CEQ) are two of the most widely used, validated instruments to probe subjective effects of psychedelic drugs. However, these assessments are lengthy and can be a burden to study participants or patients if administered during acute psychedelic effects, after resolution of psychedelic effects when participants are often fatigued, or in studies with multiple other assessments. The development of briefer measures can advance research and patient assessment.

**Methods and Materials:**

This study developed and assessed the validity of brief versions of the MEQ-30 and CEQ (MEQ-4 and CEQ-7) using data collected online from individuals reporting psychedelic use with therapeutic intent in nonstudy settings (*N* = 1160). Respondents completed full and brief versions as well as mood questionnaires indexing mental health symptoms before their psychedelic experience and now.

**Results:**

Full and brief version total scores showed strong correspondence for the MEQ (*r* = 0.89) and CEQ (*r* = 0.90). Brief versions also showed strong correspondence to full-scale subscale scores. MEQ and CEQ scores were higher for classic psychedelics (e.g., lysergic acid diethylamide, psilocybin) than for 3,4-Methylene-dioxymethamphetamine in this sample with both full and brief versions, consistent with previous full-version findings showing drug and dose-related differences using the MEQ. Also consistent with prior findings, higher mystical experience scores on both full and brief MEQ versions were associated with greater reductions in depression and anxiety, whereas challenging experiences on both full and brief CEQ versions showed limited association with changes in mental health variables.

**Conclusion:**

The notably strong association of brief scales with full versions combined with associations with therapeutic outcomes provide initial support for the MEQ-4 and CEQ-7. These findings, combined with substantial reductions in participant/patient burden, support the use of the MEQ-4 and CEQ-7 for a wide variety of research and patient treatment settings.

## Introduction

Research on serotonin 2A receptor (5-HT_2A_R) agonist (classic) psychedelics such as psilocybin and lysergic acid diethylamide (LSD) has shown initial therapeutic potential in the treatment of diverse health conditions including major depression,^1–5^ substance use disorders,^6–9^ and existential distress in patients with serious illness^10–13^ Preliminary evidence suggests that features of psychedelics' subjective effects, such as mystical-type experience (characterized by feelings of unity, positive mood, transcending time and space, and ineffability) may be associated with therapeutic outcomes^6,11,12,14,15^ and sustained benefits in healthy volunteers.^16–19^

Classic psychedelics are also known to produce intense challenging effects that can be associated with acute risks and potential harms in unsafe settings, often colloquially referred to as “bad trips.”^20^ Relevant to note, however, is that these effects and their association with clinical variables have not been found consistently across studies and that factors like the nature and setting of the drug experience as well as the dose and the specific compound(s) administered can moderate these outcomes, emphasizing the importance of measurement in varied contexts.^4,5,20–22^

This purported unique phenomenological nature of psychedelic drug effects has resulted in the development of novel instruments to probe subjective experiences within broader goals of identifying mechanisms that may underlie clinical efficacy and evaluating patient experience in clinical settings. The Mystical Experience Questionnaire (MEQ-30) and Challenging Effects Questionnaire (CEQ) are two of the most widely used instruments that have been validated and developed for use in research and clinical settings to probe features of classic psychedelics' subjective effects.^23–25^ While these scales have proven useful for clinical trials as described above, their length presents barriers to implementation into novel areas of psychedelic science.

Specifically, the 30-item MEQ-30 and 26-item CEQ may present undue participant burden when repeated administration is needed (e.g., throughout the timecourse of acute drug effects) or in any studies with multiple time-consuming assessments. Completing these instruments even once may be a burden, for example, when completed after an exhausting psychedelic administration session. Measurement in such conditions may serve to reduce validity due to participant fatigue leading to engagement in potentially problematic patterns of response bias (e.g., survey “straightlining” in which respondents select the same response every time to reduce survey duration). The development of brief measures can benefit research conducted in these settings as well as novel arenas in which rapid data collection may be needed (e.g., in large epidemiological surveys in which psychedelic subjective effects are not the primary focus; in ecological momentary assessment [EMA] studies in which participants complete multiple assessments per day remotely in their daily life).^26^

The purpose of this study was to develop and assess the validity of brief versions of the MEQ-30 and CEQ (the MEQ-4 and CEQ-7) using data collected online from individuals reporting psychedelic use with therapeutic intent in nonstudy settings. Data were collected as a part of a study evaluating people who had used a psychedelic with the intention to address psychiatric health symptoms related to anxiety, depression, and/or posttraumatic stress disorder (PTSD) or trauma, regardless of whether this psychedelic experience helped. Respondents completed both full and brief versions of the MEQ and CEQ for their most impactful psychedelic experience. Correspondence between full and brief versions was evaluated as well as the association of scale scores with psychedelic used and changes in mental health.

## Materials and Methods

### Study design and procedure

Respondents were recruited online over 1 year between July 20, 2021 and July 20, 2022 for an anonymous, cross-sectional online survey study about the effects of psychedelic use on mental health symptoms. Recruitment advertisements were placed online in forums (e.g., erowid.org) and social media platforms (e.g., twitter.com) where potential respondents could be directed to further study information and/or enroll. Study advertisements targeted individuals who had used psychedelics to help with anxiety, depression, and/or PTSD or trauma symptoms (nonmutually exclusive) regardless of whether the experience affected those symptoms.

Inclusion criteria were English-speaking adults (≥18 years) who self-reported intentional use of a classic psychedelic (e.g., ayahuasca, mushrooms, LSD, and dimethyltryptamine [DMT]) or 3,4-Methylene-dioxymethamphetamine (MDMA) to treat a mental health symptom (i.e., depression, anxiety, and PTSD/trauma). Data were collected via Qualtrics, a secure website approved for use in research. The study was reviewed and acknowledged as exempt research by a Johns Hopkins University School of Medicine Institutional Review Board because no personally identifiable information was collected.

### Measures

#### MEQ-30 and MEQ-4

The MEQ-30 consisted of 30 items completed on a 6-point scale (0 = “none; not at all,” 1 = “so slight cannot decide,” 2 = “slight,” 3 = “moderate,” 4 = “strong,” and 5 = “extreme”). The MEQ-30 includes four subscale factors: mystical, positive mood, transcendence of time and space, and ineffability. The MEQ-4 used this same 6-point scale with four items each measuring one of the four subscales (i.e., Item 1 = “sense of oneness, insight into ultimate reality, or sacredness,” Item 2 = “positive mood,” Item 3 = “transcendence of time and space,” and Item 4 = “ineffability [i.e., incapable of being expressed or described in words]”; the full measure is contained in the Supplementary Data) resulting in a 87% reduction in the number of items. Respondents were randomized to complete either the full or the brief version of the MEQ first.

#### CEQ and CEQ-7

The CEQ consisted of 26 items completed on the same 6-point scale as the MEQ. The CEQ consists of seven subscale factors: fear, grief, physical distress, insanity, isolation, death, and paranoia. The CEQ-7 used the same 6-point scale as the MEQ-30 with seven items each measuring one of the subscales using the same language as the subscale factors (e.g., “fear,” “grief”; the full measure is contained in the Supplementary Data) resulting in a 73% reduction in the number of items. Respondents were randomized to complete either the full or the brief version of the CEQ first.

#### Mental health questionnaires

All respondents completed mood questionnaires indexing mental health symptoms before their psychedelic experience and now. Respondents completed the Patient Health Questionnaire-9 (PHQ-9) as a brief measure of depressive symptoms^27^ and General Anxiety Disorder-7 (GAD-7) as a brief measure of anxiety symptoms.^28^ Respondents with a history of trauma also completed the PTSD Checklist for Diagnostic and Statistical Manual of Mental Disorders, Fifth Edition (PCL5); however, those data are not included here given that only a subset of respondents completed these measures (i.e., only those with trauma history). These mental health measures were completed in a randomized order evaluating the symptoms experienced in the two weeks before their most impactful psychedelic experience and in the two weeks before survey completion.

### Data analysis

Total score for both scales was calculated as the average of individual item scores. Although our instrument did not allow for the skipping of an individual item, for both scales, if future use entails missing items, we recommend against the calculation of a total score. Data were first evaluated for systematicity and completeness. Respondents were removed if they provided inconsistent responses (e.g., ages that differed throughout the survey, failing to report an intention to address mental health concerns in a later question), reported technical problems with the survey, or did not have complete data on the MEQ or CEQ full or brief versions. This resulted in a final sample size of 1160 respondents.^†^ Mental health data were evaluated as the arithmetic difference in symptom scores from before the psychedelic experience to currently. Correspondence between full (MEQ-30 and CEQ) and brief (MEQ-4 and CEQ-7) versions was evaluated using bivariate correlations and plotted as scatterplots with visual inspection against a 1:1 correspondence.

Differences by primary psychedelic type were conducted by comparing scores for respondents reporting a single substance used during their experience across the five most commonly reported psychedelics (i.e., psilocybin, LSD, MDMA, ayahuasca, and DMT) using a one-way analysis of variance with *post hoc* comparisons using a false discovery rate correction. Finally, clinical relevance was evaluated by evaluating the correlation between total scores on the full and brief MEQ and CEQ with changes in depression and anxiety symptoms. Analyses used *R* Statistical Language and GraphPad Prism.

## Results

### Sample and experience characteristics

Respondents (*N* = 1160) were on average 36.7 years old (standard deviation [SD] = 13.6) with 55% identifying as male, 40% as female, and 5% as neither male nor female (e.g., nonbinary). A majority of respondents were White (81.2%) and were residents of the United States (72.7%). Depression was the most commonly endorsed symptom that respondents intended to address in their psychedelic experience (82.9%) followed by anxiety (76.7%), and PTSD or trauma (58.8%) with the majority of respondents reporting multiple symptoms (76.8%).

Clinical measure scores before the psychedelic experience were 15.0 (SD = 7.5) for the PHQ-9, 12.3 (SD = 6.2) for the GAD-7, and 49.2 (SD = 18.7) for the PCL5. Clinical measures scores were currently 10.1 (SD = 7.3) for the PHQ-9, 8.2 (SD = 6.2) for the GAD-7, and 32.6 (SD = 21.1) for the PCL5. Psilocybin was the most commonly endorsed drug used during that experience (49.7%) followed by LSD (19.1%), MDMA (6.6%), ayahuasca (5.0%), and DMT (3.4%) among respondents that used only one substance. Multiple substances were endorsed by 8.6% of respondents. The median time of survey completion was 55.8 min (interquartile range = 38.9–84.0 min).

### Correspondence between MEQ-30 and MEQ-4

**FIG. 1.**
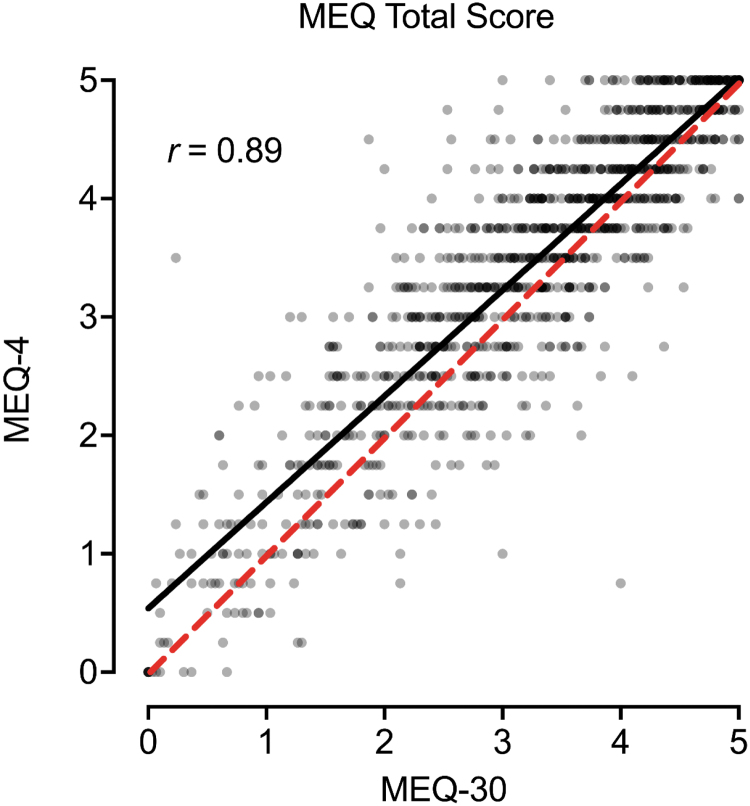
Association between MEQ-30 and MEQ-4 Total Scores. Plotted are individual data points and best fit line (black) between total scores on the MEQ-30 (full) and MEQ-4 (brief). Dotted red line is 1:1 correspondence. MEQ, Mystical Experience Questionnaire.

**FIG. 2.**
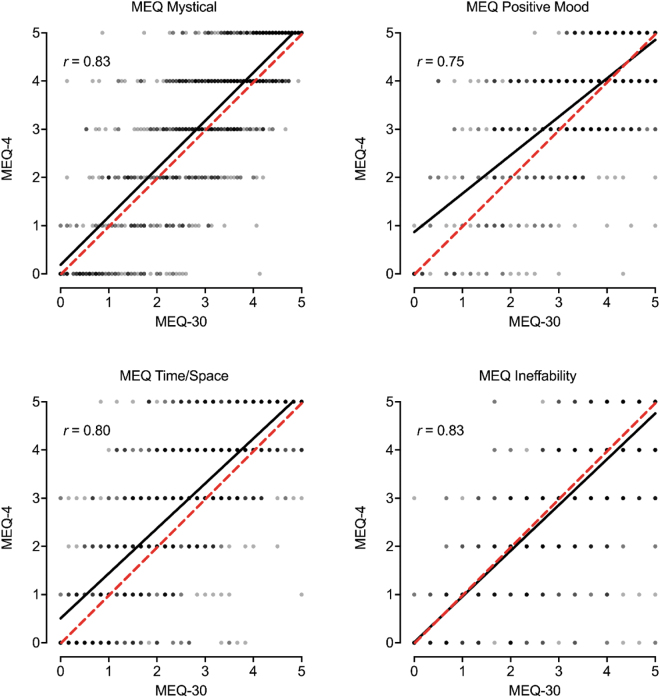
Association between MEQ-30 and MEQ-4 Subscale Scores. Plotted are individual data points and best fit line (black) between each MEQ-30 (full) subscale and corresponding MEQ-4 (brief) individual items. Dotted red line is 1:1 correspondence.

Figure 1 contains the scatterplot between the MEQ-30 and MEQ-4 total item score. A significant, positive, and strong correspondence was observed between total scores from each measure (Fig. 1; *r* = 0.89, *p* < 0.001). Scores on the MEQ-30 were modestly lower than scores on the MEQ-4 (*p* < 0.001; *d* = 0.36), although this discrepancy was consistent across the distribution of scores (Fig. 1). Similar correspondence was observed for the four MEQ subscales (Fig. 2). Table 1 contains pairwise correlations between MEQ-30 and MEQ-4 total scores and subscales within and between measures. The strongest correspondence for each total and subscale score was across measures (i.e., between MEQ-30 and MEQ-4 scores for a particular subscale score, *r* values 0.75–0.89; the bolded diagonal in Table 1). Evaluation of results by completion order did not show a differing pattern of results for the MEQ (Supplementary Data).

**Table 1.** Correlations Between MEQ-30 and MEQ-4 for Total Score and Subscales

		***MEQ-30 (full)***				
		***Total***	***Mystical***	***Positive mood***	***Time/space***	***Ineffability***
MEQ-4 (brief)	Total	**0.89**	0.84	0.74	0.74	0.71
	Mystical	0.79	**0.83**	0.60	0.57	0.49
	Positive mood	0.50	0.46	**0.75**	0.23	0.31
	Time/space	0.76	0.70	0.49	**0.80**	0.55
	Ineffability	0.67	0.57	0.50	0.61	**0.83**

Bold values represent correlation for corresponding total or subscale items.

MEQ, Mystical Experience Questionnaire.

### Correspondence between CEQ and CEQ-7

**FIG. 3.**
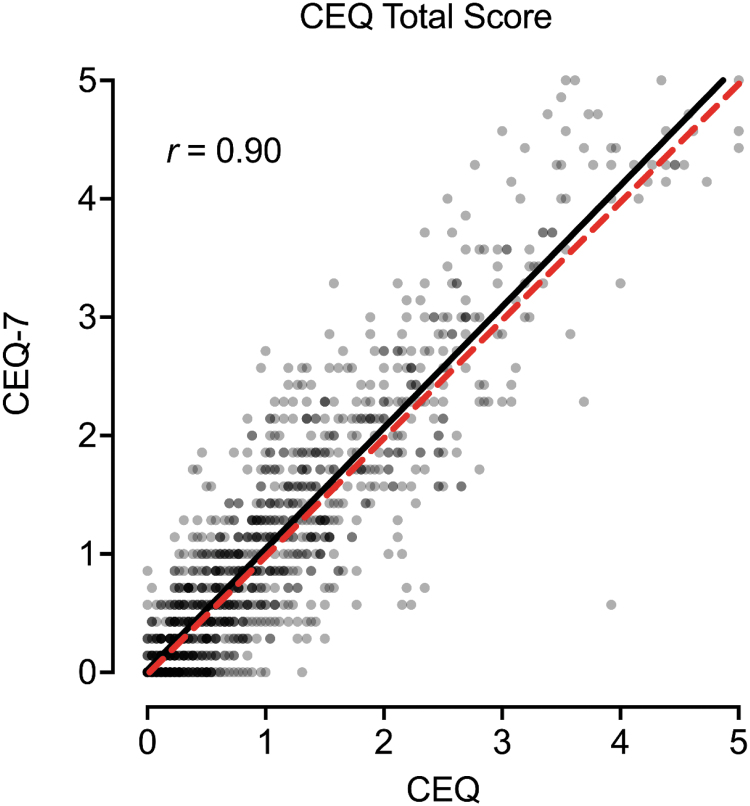
Association between CEQ and CEQ-7 Total Scores. Plotted are individual data points and best fit line (black) between total scores on the CEQ (full) and CEQ-7 (brief). Dotted red line is 1:1 correspondence. CEQ, Challenging Effects Questionnaire.

**FIG. 4.**
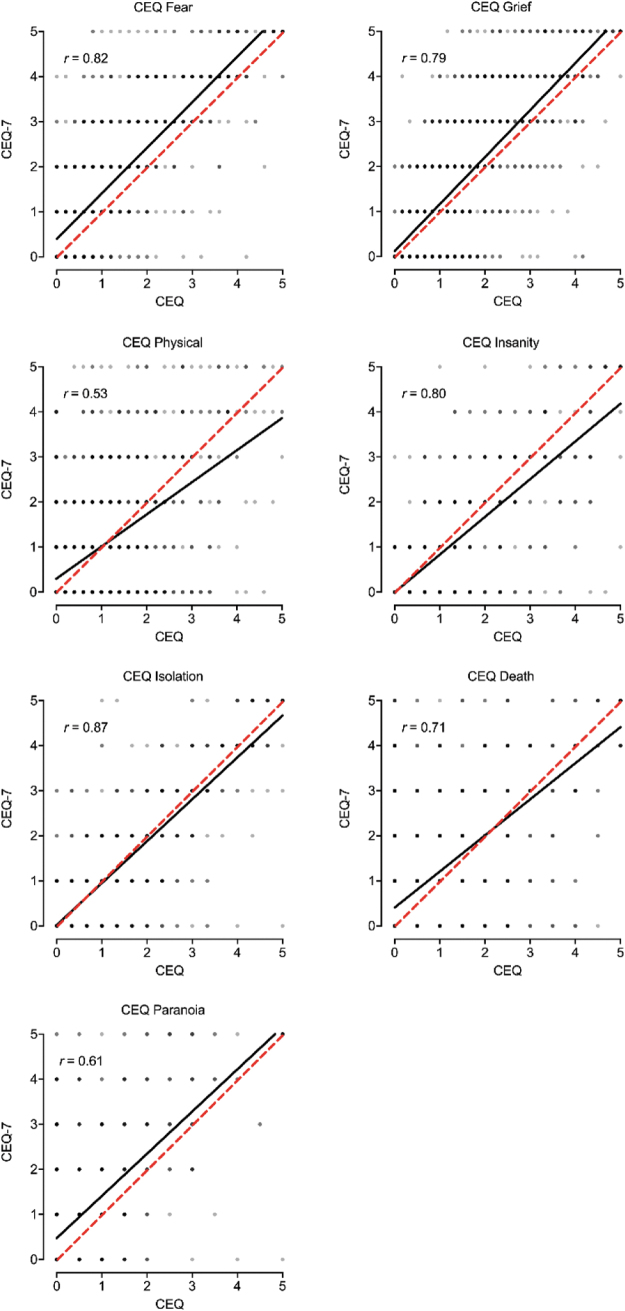
Association between CEQ and CEQ-7 Subscale Scores. Plotted are individual data points and best fit line (black) between each CEQ (full) subscale and CEQ-7 (brief) individual items. Dotted red line is 1:1 correspondence.

Figure 3 contains the scatterplot between the CEQ and CEQ-7 total item score. A significant, positive, and strong correspondence was observed between total scores from each measure (Fig. 3; *r* = 0.90, *p* < 0.001). Scores on the CEQ were significantly lower than scores on the CEQ-7 (*p* = 0.002; *d* = 0.09), although this difference was less-than-small in effect size (Cohen, 1988). Similar correspondence was observed for the seven CEQ subscales (Fig. 4). Table 2 contains pairwise correlations between CEQ and CEQ-7 total scores and subscales within and between measures. Generally, the strongest correspondence for each total and subscale score was across measures (i.e., between CEQ and CEQ-7 scores) with good correspondence observed overall (*r* values 0.53–0.90; the bolded diagonal in Table 2). Evaluation of results by completion order did not show a differing pattern of results for the CEQ (Supplementary Data).

**Table 2.** Correlations Between CEQ and CEQ-7 for Total Score and Subscales

		***CEQ (full)***							
		***Total***	***Fear***	***Grief***	***Physical***	***Insanity***	***Isolation***	***Death***	***Paranoia***
CEQ-7 (brief)	Total	**0.90**	0.83	0.76	0.59	0.74	0.70	0.57	0.57
	Fear	0.73	**0.82**	0.57	0.46	0.59	0.46	0.43	0.42
	Grief	0.60	0.45	**0.79**	0.37	0.30	0.42	0.27	0.25
	Physical	0.67	0.62	0.58	**0.53**	0.49	0.44	0.38	0.41
	Insanity	0.68	0.62	0.45	0.44	**0.80**	0.50	0.46	0.48
	Isolation	0.72	0.58	0.61	0.39	0.52	**0.87**	0.36	0.48
	Death	0.65	0.56	0.46	0.45	0.56	0.48	**0.71**	0.35
	Paranoia	0.66	0.68	0.43	0.45	0.63	0.48	0.38	**0.61**

Bold values represent correlation for corresponding total or subscale items.

CEQ, Challenging Effects Questionnaire.

### MEQ and CEQ scores by psychedelic type

**FIG. 5.**
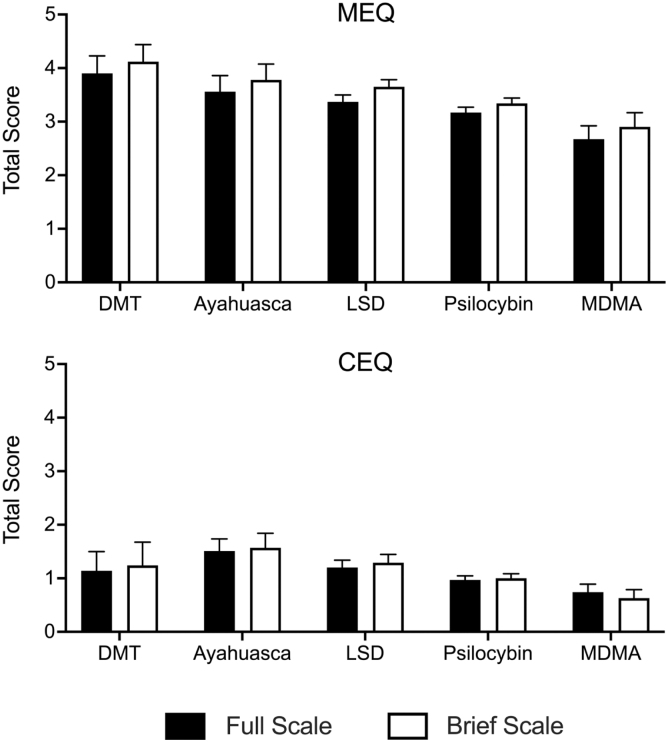
MEQ and CEQ Scores by Psychedelic Used. Plotted are MEQ and CEQ scores (mean +95% confidence interval) for the full (black bars) and brief (open bars) versions. Values are plotted based on the psychedelic used in the described experience. Data are only presented for respondents who reported using a single psychedelic in that experience. DMT, dimethyltryptamine; LSD, lysergic acid diethylamide; MDMA, 3,4-Methylene-dioxymethamphetamine.

Figure 5 contains MEQ and CEQ scores by different psychedelic used for respondents reporting only one substance used during their experience. For the MEQ-30, all drugs were significantly different from one another with the exception that ayahuasca did not significantly differ from LSD (DMT > ayahuasca = LSD > psilocybin > MDMA; see Supplementary Table S3 for pairwise comparisons). For the CEQ, all drugs were significantly different from one another with the exception that DMT did not significantly differ from LSD or psilocybin (ayahuasca > LSD = DMT = psilocybin > MDMA; see Supplementary Table S3 for pairwise comparisons). Patterns of association were similar for the brief version (white bars in Fig. 5).

### Association of MEQ and CEQ full and brief with clinical outcomes

**FIG. 6.**
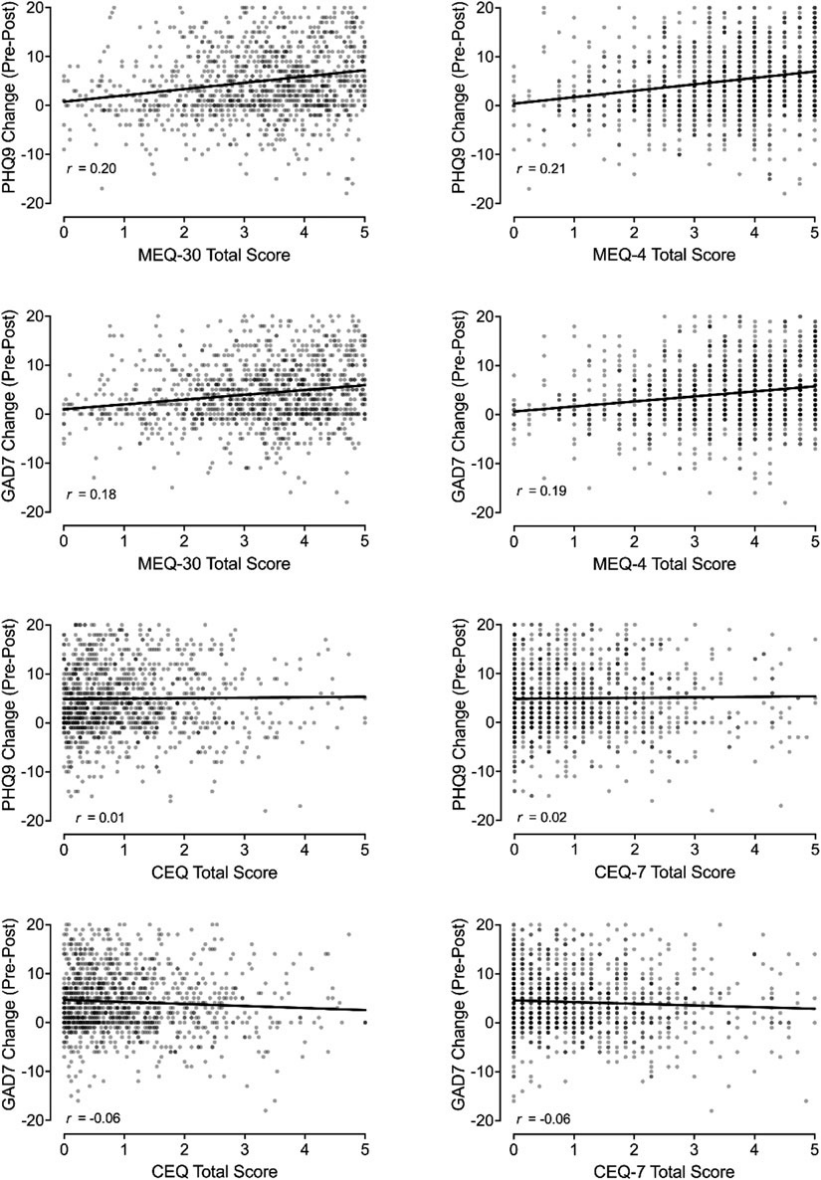
Association of MEQ and CEQ Scores with Changes in Depression and Anxiety. Plotted in left panels are associations involving the MEQ-30 and CEQ (full scales). Plotted in right panels are associations involving the MEQ-4 and CEQ-7 (brief scales). *x*-Axes depict changes in PHQ-9 (depression) and GAD-7 (anxiety) scores from before psychedelic experience to now.

Figure 6 contains the association between MEQ scores and changes in depressive (PHQ-9) and anxiety (GAD-7) symptoms. These correlations were all significant and small-to-medium in effect size (*r* values = 0.18–0.21, *p* < 0.001) indicating that greater mystical experience scores were associated with a greater reduction in depression and anxiety symptoms from before the most impactful psychedelic experience until now. Figure 6 also contains the association between CEQ scores and changes in depressive and anxiety symptoms. Associations between changes in anxiety and CEQ (*r* = −0.06, *p* = 0.034) and CEQ-7 (*r* = −0.06, *p* = 0.048) were significant, but less than small in effect size (Cohen, 1988). The correlation between changes in depressive symptoms and either the CEQ or the CEQ-7 total score were not significant.

## Discussion

This study evaluated brief versions of the MEQ-30 and CEQ for measuring psychedelic drug effects. Brief versions of these measures were designed to mirror the underlying subscales of the MEQ-30 and CEQ resulting in four and seven item measures and item number reductions of 87% and 73%, respectively. Comparison of full and brief versions demonstrated a close correspondence for total scores. Brief versions also showed strong correspondence to full-scale subscale scores. Additional results show good correspondence to existing psychedelic literature using the full scales and clinical expectations. MEQ and CEQ scores were higher for classic psychedelics than MDMA for both versions. Higher MEQ scores on full and brief versions were associated with greater reductions in depression and anxiety. In contrast, CEQ scores showed limited association with changes in mental health variables, consistent with recent clinical trial data and other observational datasets.^4,29,30^ All of these findings provide strong initial support for the use of the MEQ-4 and CEQ-7 in broad psychedelic science and related research domains.

The goal of the MEQ-4 and CEQ-7 is to provide a method for rapidly assessing the subjective experience of psychedelic drug effects under conditions in which full-scale responses may be pragmatically prohibitive and burdensome to participants/patients. To date, evaluation of the MEQ-30 and CEQ has largely been constrained to retrospective recall over the course of an experience, likely stemming from the burdensome nature of these longer inventories. The use of brief measures would allow for collection of drug effects throughout a full timecourse of drug action thus affording the opportunity to evaluate novel questions regarding the onset and offset of specific features of the drug experience and possible correspondence with clinical benefit.

Likewise, brief measures can allow for expansion of measurement into novel settings such as in large epidemiological surveys in which psychedelic subjective effects are not the primary focus, and ambulatory settings via EMA. For example, implementation of brief measures in large-scale epidemiological projects like the National Survey on Drug Use and Health may better inform the public health impact of naturalistic psychedelic use.^31^ Such research in real-world or ambulatory settings would allow for the determination of not only the acute effects of psychedelics but also how variations in person–place interactions influence subjective experiences of psychedelic drugs (e.g., Ref.^32^). The strong overlap of brief with full versions reported here supports these varied applications. Moreover, we and other scientists who have conducted numerous psychedelic administration studies can attest to the participant burden of completing numerous lengthy scales even in the traditional end-of-session use for such scales. Participants often report feeling physically and mentally exhausted after such sessions. The strong correspondence observed in this study suggests that even for this traditional end-of-session use, the brief versions of these scales may constitute a substantial advantage.

Scores for the full and brief MEQ versions were higher for classic psychedelics than for MDMA with a ranked order of DMT > ayahuasca = LSD > psilocybin > MDMA. These results are broadly consistent with prior work documenting higher MEQ-30 scores for participants receiving classic psychedelics compared to MDMA (e.g., Refs.^33,34^). Similarly, CEQ scores were highest for ayahuasca and lowest for MDMA.

Although we are not aware of laboratory research that has directly compared CEQ scores for MDMA to classic psychedelics (see systematic review of adverse event reporting in Ref.^35^), these findings align with the high rates of negative symptoms such as gastrointestinal distress and nausea reported with ayahuasca use (e.g., Refs.^36,37^), and are generally consistent with the notion that classic psychedelics are often more anxiety-provoking than MDMA (e.g., Ref.^38^). While the mechanistic rationale for differences observed between classic psychedelics and MDMA is not fully delineated (e.g., differences in primary receptor activity and monoaminergic effect profiles), these results more proximally support the ability of the MEQ-4 and CEQ-7 to recapitulate expected outcomes by drug type, further emphasizing measure validity.

Changes in mental health symptoms were associated with MEQ scores, but not CEQ scores. Several studies have demonstrated a similar relationship between higher mystical experience scores and improved therapeutic outcomes following psychedelic-assisted treatment.^6,11,12,14–19^ Although the CEQ has been less utilized in therapeutic trials, recent studies suggest it is not correlated with persisting clinical outcomes (e.g., Refs.^4,39^). These findings further emphasize measure validity by replicating clinical results and, more broadly, is suggestive of a possible clinical usefulness of brief measures that is akin to that attributed to full measures.

Significant criticism of the relevance of subjective experiences for therapeutic drug effects has been raised with debate regarding whether subjective experience is necessary for therapeutic drug effects or may be epiphenomenal to other changes (e.g., neurotropic or neuroplastic changes) mediating positive clinical outcomes.^40,41^ Brief measures may allow for additional opportunities to test these hypotheses by allowing for more inclusive measurement in clinical protocols and in potential future nonresearch clinical care settings.

It is also helpful to contextualize the MEQ-4 and CEQ-7 with regard to other existing measures. In addition to the MEQ30 and CEQ, a number of instruments have been used to assess features of drug-induced altered states of consciousness.^42^ These measures attempt to capture notable qualities of psychedelics' subjective effects, which may in turn be useful for predicting therapeutic response, providing warnings of potential harms, and otherwise understanding psychological mechanisms of psychedelic drug actions in therapeutic and other (e.g., spiritual, recreational) settings. With the large, and growing, number of variables of interest at play in psychedelic research and therapies, our hope is that the MEQ-4 and CEQ-7 can provide a streamlined means of assessing integral subjective drug effects while allowing for increased implementation during acute and postacute drug administration to better characterize these qualities over time while also minimizing participant burden.

This initial study collected data via a web-based assessment method, which means that we relied on recall of subjective experiences with limited control over the measurement environment or context of the psychedelic experience (c.f., measurement after controlled laboratory dosing). We took significant steps to ensure data quality including rigorous screening of data for consistency of responding to improve the fidelity of data collected.

One potential concern is that these data relied on retrospective recall of one's experience with psychedelics as well as recall of psychological health and well-being, which could impact responding depending on the delay and quality of this recall. Relatedly, for the evaluation of clinical utility, we focused on a measure of change in mental health symptoms from before the psychedelic experience to now, which may vary depending on the time since the attributed experience. Supplemental analyses (Supplementary Fig. S1) indicated no difference in MEQ or CEQ full or brief scores based on the time since a respondent's recalled psychedelic experience, however, partly mitigating concerns regarding this recall.

## Conclusions

This study introduced and evaluated two brief measures for measuring psychedelic drug effects—the MEQ-4 and Challenging Experience Questionnaire-7 (CEQ-7). Both the MEQ-4 and CEQ-7 showed robust correspondence with full item counterparts as well as expected associations with drug-related and clinical variables supporting the validity of these brief measures. Brief measures will advance psychedelic science and clinical practice by allowing for measurement in a wider variety of contexts and with less participant/patient burden, affording the opportunity to evaluate novel questions underlying psychedelic drug effects and therapeutic mechanisms.

## Notes

^†^
Nonexclusive reasons for exclusion were reporting mismatching ages (*n* = 70), inconsistent reporting of psychedelic intentions (*n* = 289), technical problems with the survey (*n* = 11), and missing MEQ/CEQ data (*n* = 57).
