# Supplementary material for: The Mystical Experience Questionnaire 4-Item and Challenging Experience Questionnaire 7-Item
Source: Psychedelic Med (New Rochelle). 2024 Mar 12;2(1):33–43. doi: 10.1089/psymed.2023.0046 (PMC11658653; doi:10.1089/psymed.2023.0046)
Supplement: Supplementary Data [file psymed.2023.0046_suppl_data.docx]

**Supplemental Materials**

**Mystical Experience Scale Brief (MEQ-4)**

Looking back on the entirety of your experience, please rate the degree to which at any time you experienced the following phenomena. Answer each question according to your feelings, thoughts, and experiences at the time of the session. In making each of your ratings, use the following scale:

-None/Not at all

-So slight cannot decide

-Slight

-Moderate

-Strong (equivalent in degree to any other strong experience)

-Extreme (more than any other time in my life and stronger than "strong")

1. Sense of oneness, insight into ultimate reality, or sacredness
2. Positive mood
3. Transcendence of time and space
4. Ineffability (i.e., incapable of being expressed or described in words)

**Challenging Experience Questionnaire Brief (CEQ-7)**

Looking back on the entirety of your experience, please rate the degree to which at any time you experienced the following phenomena. Answer each question according to your feelings, thoughts, and experiences at the time of the session. In making each of your ratings, use the following scale:

-None/Not at all

-So slight cannot decide

-Slight

-Moderate

-Strong (equivalent in degree to any other strong experience)

-Extreme (more than any other time in my life and stronger than "strong")

1. Fear
2. Grief
3. Physical distress
4. Insanity
5. Isolation
6. Thoughts or feelings of death
7. Paranoia

‘

Supplemental Table 1. Correlations between MEQ-30 and MEQ-4 for Total Score and Subscales Based on Completion Order

|  |  | MEQ-30 (Full) | | | | |
| --- | --- | --- | --- | --- | --- | --- |
|  |  | Total | Mystical | Pos. Mood | Time/Space | Ineffability |
|  |  | Brief Scale First | | | | |
| MEQ-4 (Brief) | Total | 0.86 | 0.80 | 0.73 | 0.72 | 0.70 |
|  | Mystical | 0.76 | 0.78 | 0.61 | 0.55 | 0.47 |
|  | Pos. Mood | 0.47 | 0.43 | 0.71 | 0.22 | 0.27 |
|  | Time/Space | 0.75 | 0.70 | 0.51 | 0.76 | 0.55 |
|  | Ineffability | 0.65 | 0.54 | 0.47 | 0.63 | 0.80 |
|  |  | Brief Scale Second | | | | |
|  | Total | 0.92 | 0.87 | 0.75 | 0.76 | 0.74 |
|  | Mystical | 0.84 | 0.88 | 0.60 | 0.60 | 0.53 |
|  | Pos. Mood | 0.53 | 0.48 | 0.78 | 0.25 | 0.34 |
|  | Time/Space | 0.77 | 0.70 | 0.47 | 0.85 | 0.57 |
|  | Ineffability | 0.69 | 0.60 | 0.54 | 0.59 | 0.86 |

Supplemental Table 2. Correlations between CEQ and CEQ-7 for Total Score and Subscales Based on Completion Order

|  |  | CEQ (Full) | | | | | | | |
| --- | --- | --- | --- | --- | --- | --- | --- | --- | --- |
|  |  | Total | Fear | Grief | Physical | Insanity | Isolation | Death | Paranoia |
|  |  | Brief Scale First | | | | | | | |
| CEQ-7 (Brief) | Total | 0.91 | 0.84 | 0.78 | 0.63 | 0.74 | 0.75 | 0.57 | 0.58 |
|  | Fear | 0.72 | 0.81 | 0.60 | 0.48 | 0.57 | 0.51 | 0.42 | 0.42 |
|  | Grief | 0.60 | 0.45 | 0.80 | 0.39 | 0.29 | 0.42 | 0.30 | 0.21 |
|  | Physical | 0.71 | 0.66 | 0.64 | 0.57 | 0.52 | 0.52 | 0.40 | 0.42 |
|  | Insanity | 0.66 | 0.60 | 0.45 | 0.46 | 0.80 | 0.52 | 0.46 | 0.49 |
|  | Isolation | 0.72 | 0.63 | 0.60 | 0.44 | 0.57 | 0.86 | 0.37 | 0.50 |
|  | Death | 0.66 | 0.56 | 0.49 | 0.47 | 0.57 | 0.54 | 0.66 | 0.41 |
|  | Paranoia | 0.65 | 0.69 | 0.41 | 0.46 | 0.61 | 0.52 | 0.36 | 0.64 |
|  |  | Brief Scale Second | | | | | | | |
|  | Total | 0.92 | 0.83 | 0.76 | 0.57 | 0.74 | 0.69 | 0.59 | 0.56 |
|  | Fear | 0.77 | 0.86 | 0.56 | 0.47 | 0.63 | 0.47 | 0.46 | 0.43 |
|  | Grief | 0.63 | 0.46 | 0.81 | 0.36 | 0.32 | 0.44 | 0.25 | 0.31 |
|  | Physical | 0.64 | 0.59 | 0.54 | 0.52 | 0.47 | 0.38 | 0.37 | 0.40 |
|  | Insanity | 0.69 | 0.64 | 0.45 | 0.41 | 0.81 | 0.50 | 0.47 | 0.48 |
|  | Isolation | 0.71 | 0.53 | 0.63 | 0.35 | 0.48 | 0.89 | 0.34 | 0.47 |
|  | Total | 0.64 | 0.56 | 0.43 | 0.42 | 0.56 | 0.43 | 0.77 | 0.28 |
|  | Fear | 0.68 | 0.67 | 0.45 | 0.44 | 0.65 | 0.46 | 0.41 | 0.57 |

Supplemental Table 3. Pairwise Comparisons of MEQ and CEQ Total Scores by Drug

| Comparison | MEQ-30 | MEQ-4 | CEQ | CEQ-7 |
| --- | --- | --- | --- | --- |
| Ayahuasca vs. DMT | 0.035 | 0.036 | 0.046 | 0.072 |
| Ayahuasca vs. LSD | 0.054 | 0.094 | 0.033 | 0.046 |
| Ayahuasca vs. MDMA | <0.001 | <0.001 | <0.001 | <0.001 |
| Ayahuasca vs. Psilocybin | 0.004 | 0.002 | <0.001 | <0.001 |
| DMT vs. LSD | 0.003 | 0.005 | 0.450 | 0.331 |
| DMT vs. MDMA | <0.001 | <0.001 | 0.033 | 0.003 |
| DMT vs. Psilocybin | <0.001 | <0.001 | 0.193 | 0.083 |
| LSD vs. MDMA | <0.001 | <0.001 | 0.001 | <0.001 |
| LSD vs. Psilocybin | 0.007 | <0.001 | 0.003 | 0.001 |
| MDMA vs. Psilocybin | <0.001 | 0.001 | 0.041 | 0.003 |

Note. False discovery rate corrected significant values for pairwise comparisons for each scale based on drug used during the described psychedelic experience.

Supplemental Figure 1. Association of Time Since Psychedelic Use and MEQ/CEQ Scores

**
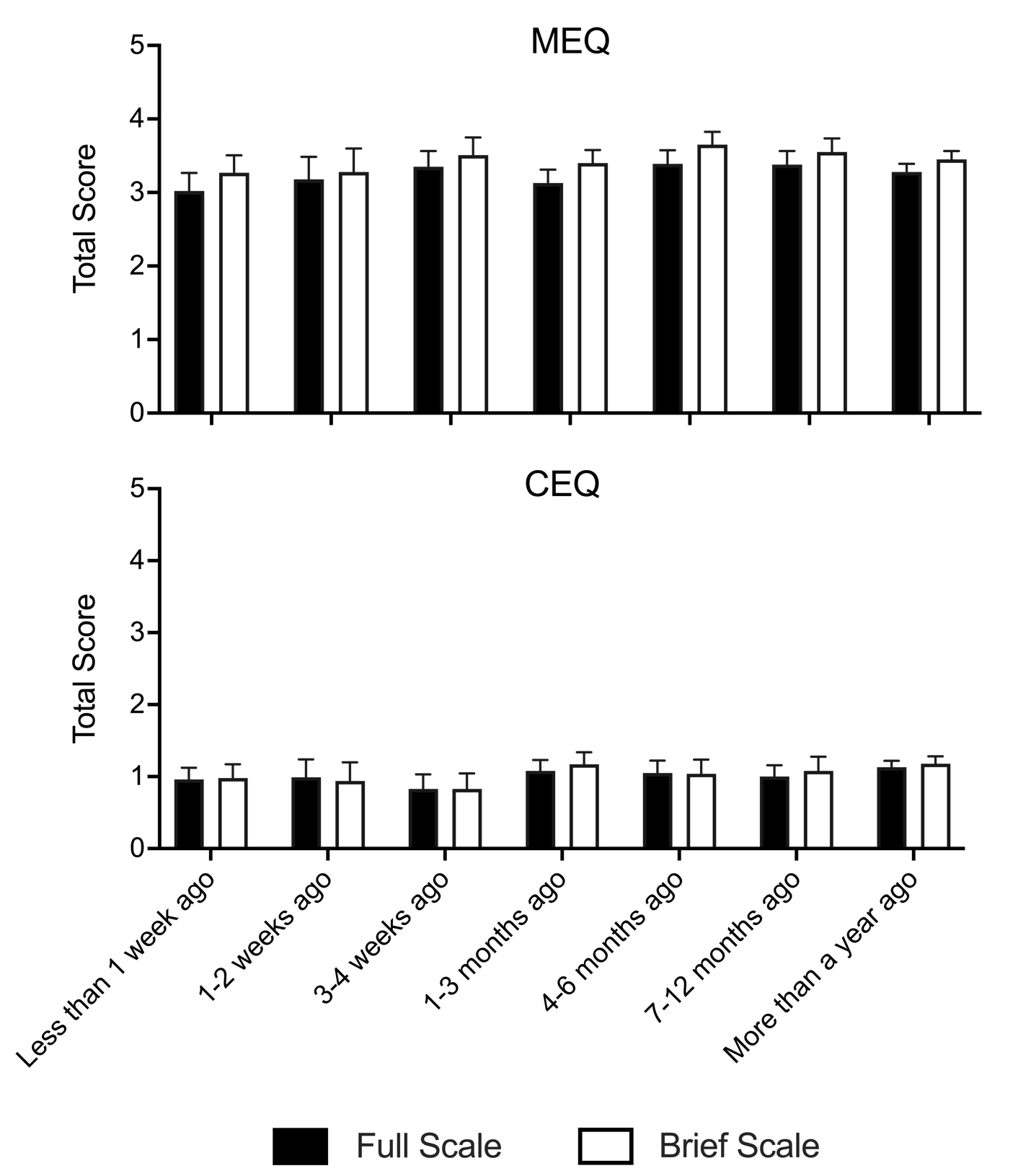
**
